# High burden of acute respiratory tract infections leading to hospitalization at German pediatric hospitals: fall/winter 2022–2023

**DOI:** 10.1007/s15010-023-02123-7

**Published:** 2023-11-13

**Authors:** Maren Doenhardt, Jakob P. Armann, Natalie Diffloth, Christin Gano, Josephine Schneider, Dominik T. Schneider, Tobias Tenenbaum, Andreas Trotter, Reinhard Berner, the DGPI Ad hoc-ARI-register working group, Martina Aderhold, Jürgen Althaus, Theresa Andree, Tobias Ankermann, Nils Apel, Stefan Arens, Ulrich Aumann, Thomas Austgen, Rebekka Baier, Lisa Baresi, Marcel Baschin, Stefanie Beil, Christiane Bell, Giulia Bender, Bernd Bergmann, Sonja Bernlochner, Silke Bettinger, Adina Biering, Leonie Block, Henning Böhme, Carsten Bölke, Timon Boßlet, Reinhard Bullmann, Iryna Dobrianska, Christoph Ehrsam, Martin Enders, Anna-Lisa Erler, Inge Evers, Andreas Fiedler, Elisa Fingerloos, Veronika Galajda Pavlíková, Lars Geerdts, Katharina Glas, Julia Gottschalk, Anne Grimm, Katrin Gröger, Volkmar Grosse, Anneke Grotheer, Katharina Hecker, Maik Heine, Georg Heubner, Aneta Horakova, Jennifer Horn, Daniel Hubert, Kristin Jähnert, Simone Jedwilayties, Andrea Jehn, Sumathy Jeyaweerasinkam, Jasmin Joiko, Hermann Kalhoff, Marcus Kania, Veronika Kautzky, Thomas Keller, Margit Kellerer, Mandy Kersten, Karoline kinkelin, Andreas Klein, Christof Kluthe, Louise Kobelt, Susanne Kremsreiter, Benno Kretzschmar, Merten Kriewitz, Sophia Kuhl, Gerrit Lautner, Vincenzo Leone, Klaus Lohmeier, Daniela Lubitz, Sabine Mahncke, Anja Mayer, Peter Meißner, Egbert Meyer, Marko Mohorovicic, Barbara Müksch, Barbara Naust, Michael Nippes, Esra Özdemir, Denisa Penfold, Ursula Pindur, Daniela Pütz, Anke Rappen, Friedrich Reichert, Marie-Charlotte Rosahl, Miriam Ryba, Shahana Safarova, Asimina Salapata, Christoph Schick, Anna Schlegel, Norbert Schmeja, Juliane Schmid, Robert Schmitz, Dominik Schneider, Florian Schneider, Anna Schrafstetter, Leonie Schulteß, Johannes Schulze, Kerstin Schunke, Lavinia Seidel, M Ghiath Shamdeen, Josef Sonntag, Michael Steidl, Tobias Stiefel, Julia Tabatabai, Maren Thurner, Sandra Trapp, Mirjam Ungerechts, Manja Unrath, Alijda Ingeborg van den Heuvel, Kai Vehse, Joshua Verleysdonk, Christian von Schnakenburg, Simone Wagner, Andreas Wemhöner, Barbara Wichmann, Christiane Maria Wiethoff, Thomas Wollbrink, Andreas Wroblewski, Ulrich Zügge

**Affiliations:** 1 Department of Pediatrics, University Hospital and Medical Faculty Carl Gustav Carus, Technische Universität Dresden, Fetscherstraße 74, 01307 Dresden, Germany; 2 Clinic of Pediatrics, Municipal Hospital Dortmund, University Witten/Herdecke, Beurhausstraße 40, 44137 Dortmund, Germany; 3 Clinic for Child and Adolescent Medicine, Sana Klinikum Lichtenberg, Academic Teaching Hospital, Charité-Universitätsmedizin Berlin, Fanningerstraße 32, 10365 Berlin, Germany; 4 Children’s Hospital and Center for Perinatal Medicine, Teaching Hospital of the University of Freiburg, Virchowstr. 10, 78224 Singen, Germany

**Keywords:** Acute respiratory infection, RSV, Influenza, SARS-CoV-2, Children, Hospitalization

## Abstract

**Purpose:**

Given reduced immunity levels for seasonally occurring respiratory infections and the experience of an unusually early, severe wave of RSV infections during 2021, a preexisting clinician-led reporting system (CLRS) was updated to prospectively monitor the anticipated high burden of respiratory infections (ARI) in German pediatric hospitals during fall/winter 2022–2023.

**Methods:**

From September 13, 2022 through March 31, 2023, children hospitalized with ARI as a primary diagnosis were monitored via a national CLRS established by the German Society for Pediatric Infectious Diseases (DGPI). Once a week, the CLRS collected overall number of new hospital admissions, ARI-related admissions according to pathogen (SARS-CoV-2, RSV, influenza, and other), plus number of patients admitted to ICU with ARI as a primary diagnosis.

**Results:**

With a high participation among children's hospitals across Germany (22.8%), 76 centers submitted 1,053 survey reports. ARI-related hospital admissions showed a steep rise starting in late September 2022 and reached their highpoint in early December 2022 (50.1% of all admissions). In parallel, the average number of newly admitted patients (aNA) with RSV (3.6) peaked, as did those with influenza (2.1) one week later. The average highpoint of ARI patients on ICU (aICU) (2.9) was reached shortly thereafter. Again, RSV (1.6) und influenza (1.2) were predominant pathogens.

**Conclusion:**

In fall/winter 2022–2023, German hospitals reported a sharp increase in patients with ARIs. While RSV and influenza represented the greatest proportion of ARI, SARS-CoV-2 played a less significant role. Systematic, dynamic collection of ARI data is critical for assessing real burdens on the health care system.

**Supplementary Information:**

The online version contains supplementary material available at 10.1007/s15010-023-02123-7.

## Introduction

As a result of the COVID-19 pandemic-related implementation of non-pharmaceutical public health measures, such as masking and quarantining, infections with non-SARS-CoV-2-related acute respiratory pathogens became less frequent during the first year of the pandemic (2020) versus pre-pandemic years [1]. This decline in acute respiratory infections (ARI) led to immunity gaps for other respiratory viruses [2], a development that ultimately resulted in an early and particularly severe RSV season for the pediatric population during fall/winter 2021–2022 [3]. The RSV burden during the fall/winter 2021–2022 season in Germany was captured by a clinician-led reporting system (CLRS) established by the German Society for Pediatric Infectious Diseases (DGPI) [3, 4]. This same system also was used to collect data on disease burdens during the Omicron wave in Germany in early 2022 [5, 6]. In order to assess the impact of different respiratory viruses on disease burden and hospitalizations in the pediatric population following the cessation of SARS-CoV-2-related public health measures in 2022, the DGPI expanded the CLRS’s scope in order to capture hospital admissions due not just to RSV and SARS-CoV-2, but also to other pathogens causing ARI among children and adolescents at German pediatric hospitals during the 2022–2023 season. Thus, rather than recording each individual, positive test result as was done in the initial CLRS, the modified CLRS emphasized patients' main admission diagnoses. This change in the CLRS's primary objective allowed mapping the real burden of hospitalization for ARI, thereby minimizing the impact of incidentally positive test results.

## Materials and methods

The updated version of the CLRS, launched on September 13, 2022 and active through March 31, 2023, shifted focus so as to capture data on a weekly basis, with Tuesdays selected as a regular reference reporting day. Data now collected included:

1. overall number of hospital admissions, regardless of admission diagnoses;
2. number of hospital admissions where ARI were recorded as the primary reason for admission;
3. number of patients receiving intensive care treatment who also had ARI as a primary diagnosis;
4. number of distinctly-detected respiratory pathogens having led to hospital admission and/or intensive care treatment (ICU); and
5. patient age (according to age group) for each pathogen.

With regard to ARI, the main pathogens specifically tracked by the CLRS included: SARS-CoV-2, RSV, and influenza. Survey participants additionally had the option of choosing "other confirmed respiratory pathogen". In cases where pathogen detection was pending or missing, the survey allowed participants to select "unknown pathogen". For ICU patients, the survey collected additional data how many of these patients were in need of respiratory support (invasive vs. non-invasive) and how many patients had significant comorbidities. No data were collected regarding type and severity of the existing comorbidities.

The CLRS additionally requested reporting of coinfections. CLRS guidelines for survey participants stated the following reporting procedures: When calculating total number of patients (new admissions or admissions on ICU), patients with coinfections only were to be counted once. If coinfections occurred within the group of patients being reported on a specific day, the number of patients infected with more than one pathogen should be recorded. As a follow-up to the later, in the pathogen-specific part of the survey, the survey requested that coinfections be documented separately in connection with each of the relevant pathogens detected. E.g., a two-year-old patient with a RSV and SARS-CoV-2 coinfection should be as just one hospital admission, but then in the part of the survey where RSV and SARS-CoV-2-specific data were being captured, this patient would be counted as *both* a RSV case and a SARS-CoV-2 case. Each pathogen group was considered as an individual group. Unfortunately, it is not possible for us to retrospectively match which pathogens occurred together as coinfections.

Age groups were defined as: newborns (0–3 months of life), infants (4–11 months), toddlers (1–2 years), preschool-aged children (3–4 years), school-aged children (5–11 years), adolescents (12–18 years), and young adults (≥ 19 years).

As with the initial version of the CLRS launched in 2021, the updated CLRS (launched in 2022) was promoted via the websites of the DGPI and the German Society of Pediatrics (DGKJ), as well as additionally announced in a newsletter sent to all German pediatric hospitals who regularly, voluntarily submit anonymized data on hospital admissions of children and adolescents. Data collected through the CLRS also were publicly accessible via the DGPI website [7]. Study data were collected and managed using REDCap (Research Electronic Data Capture) software, hosted at the Technical University Dresden, Germany [8, 9]. Analyses were performed using Microsoft Excel v.2010. Graphics were created using Datawrapper software (datawrapper.de).

Our descriptive statistics are presented as absolute frequencies with percentages for categorical variables. Average case numbers were calculated based upon cases reported per reporting hospital per day. They were then additionally coded as average numbers of newly admitted patients per reporting hospital per day (aNA) and as average numbers of ICU patients who had ARI as a main diagnosis per reporting hospital per day (aICU), respectively. When calculating the aICU, only hospitals with an in-house ICU were included.

Prior to initiation, the survey was reviewed and approved by the Ethics Committee of the Technical University Dresden.

## Results

### Dynamic proportions of different respiratory pathogens over time

The CLRS obtained representation from all German federal states and included both small and large hospitals from primary to tertiary care. Over the full time frame of the survey, 1,053 reports from 76 children's hospitals were submitted—a participation rate equivalent to 22.8% of all German children's hospitals. At the peak of survey participation (December 13, 2022), 53 of 334 German pediatric hospitals (15.9%) submitted reports. Lowest participation rates were in the beginning and at the end of the survey (September 13, 2022 and March 28, 2023), with 21 (6.3%) and 23 (6.9%) hospitals participating. Calculated over the whole study period, the median number of reporting hospitals was 37 (10.9%).

In total, 3245 newly admitted patients and 763 ICU patients with ARI as their main diagnosis were reported to the CLRS (Table 1).

**Table 1:** Absolute case numbers of reported patients: (a) newly admitted patients with an acute respiratory infection (ARI) as their primary admission diagnosis, and (b) ICU patients with ARI as a primary diagnosis

	All	SARS-CoV-2		RSV		Influenza		Other confirmed respiratory pathogens		Unknown respiratory pathogens^a^	
Number of individual patients	*N*	*n*/*N*	%	*n*/*N*	%	*n*/*N*	%	*n*/*N*	%	*n*/*N*	%
(a) newly admitted	**3245**^c^	**252**/3245	7.8	**1005**/3245	31.0	**444**/3245	13.7	**286**/3245	8.8	**1338**/3245	41.2
Age											
Newborns (0–3 months)		94/**252**	37.3	423/**1005**	42.8	50/**444**	11.3	30/**286**	10.5	148/**1338**	11.1
Infants (4–11 months)		65/**252**	25.8	277/**1005**	27.6	47/**444**	10.6	38/**286**	13.3	185/**1338**	13.8
Toddlers (1–2 years)		40/**252**	15.9	187/**1005**	18.6	92/**444**	20.7	88/**286**	30.8	450/**1338**	33.6
Preschool-aged children (3–4 years)		10/**252**	4.0	75/**1005**	7.4	95/**444**	21.4	66/**286**	23.1	239/**1338**	17.9
School-aged children (5–11 years)		22/**252**	8.7	22/**1005**	2.2	115/**444**	25.9	46/**286**	16.1	219/**1338**	16.4
Adolescents (12–18 years)		21/**252**	8.3	7/**1005**	0.7	38/**444**	8.6	12/**286**	4.2	45/**1338**	3.4
Young Adults (≥ 19 years)		1/**252**	0.4	0/**1005**	0.0	0/**444**	0.0	0/**286**	0.0	1/**1338**	0.1
Age not specified		0/**252**	0.0	14/**1005**	1.4	7/**444**	1.6	6/**286**	2.1	51/**1338**	3.8
(b) on intensive care unit (ICU)	**763 **^**c**^	**35**/763	4.6	**328**/763	43.0	**105**/763	13.8	**183**/763	24.0	**145**/763	19.0
Age											
Newborns (0–3 months)		8/**35**	22.9	192/**328**	58.5	14/**105**	13.3	28/**183**	15.3	23/**145**	15.9
Infants (4–11 months)		14/**35**	40.0	65/**328**	19.8	11/**105**	10.5	35/**183**	19.1	18/**145**	12.4
Toddlers (1–2 years)		1/**35**	2.9	37/**328**	11.3	26/**105**	24.8	50/**183**	27.3	33/**145**	22.8
Preschool-aged children (3–4 years)		2/**35**	5.7	13/**328**	4.0	17/**105**	16.2	20/**183**	10.9	17/**145**	11.7
School-aged children (5–11 years)		3/**35**	8.6	7/**328**	2.1	24/**105**	22.9	30/**183**	16.4	35/**145**	24.1
Adolescents (12–18 years)		5/**35**	14.3	10/**328**	3.1	11/**105**	10.5	18/**183**	9.8	15/**145**	10.3
Young Adults (≥ 19 years)		2/**35**	5.7	1/**328**	0.3	0/**105**	0.0	0/**183**	0.0	0/**145**	0.0
Age not specified		0/**35**	0.0	3/**328**	0.9	2/**105**	1.9	2/**183**	1.1	4/**145**	2.8
On ICU, including additional information on comorbidities^b^		**28/35**	80.0	**186/328**	56.7	**71/105**	67.6	**130/183**	71.0	**112/145**	77.2
On ICU with comorbidities^b^		15/**28**	53.6	63/**186**	33.9	44/**71**	62.0	75/**130**	57.7	71/**112**	63.4
On ICU, including additional information on respiratory support^b^		**30/35**	85.7	**289/328**	88.1	**89/105**	84.8	**159/183**	86.9	**131/145**	90.3
On ICU with respiratory support^b^		19/**30**	63.3	265/**289**	91.7	67/**89**	75.3	133/**159**	83.6	102/**131**	77.9
With invasive ventilation^b^		9/**30**	30.0	34/**289**	11.8	22/**89**	24.7	42/**159**	26.4	24/**131**	18.3
With non-invasive ventilation^b^		10/**30**	33.3	231/**289**	79.9	45/**89**	50.6	91/**159**	57.2	78/**131**	59.5

Age distribution is shown for each pathogen. For ICU patients, absolute numbers for those with comorbidities and/or with need for respiratory support are stated for different pathogen groups only in cases where information was submitted. (Entry of this additional information was optional.)

The text marked in bold indicates which sample was used to calculate the percentage values

^a^Pathogen detection pending/not performed

^b^Percentages are calculated in relation to absolute case numbers in reports where information was provided

^c^Due to existing coinfections, the absolute number of individual patients is lower than the overall sum of all

**Fig. 1:**
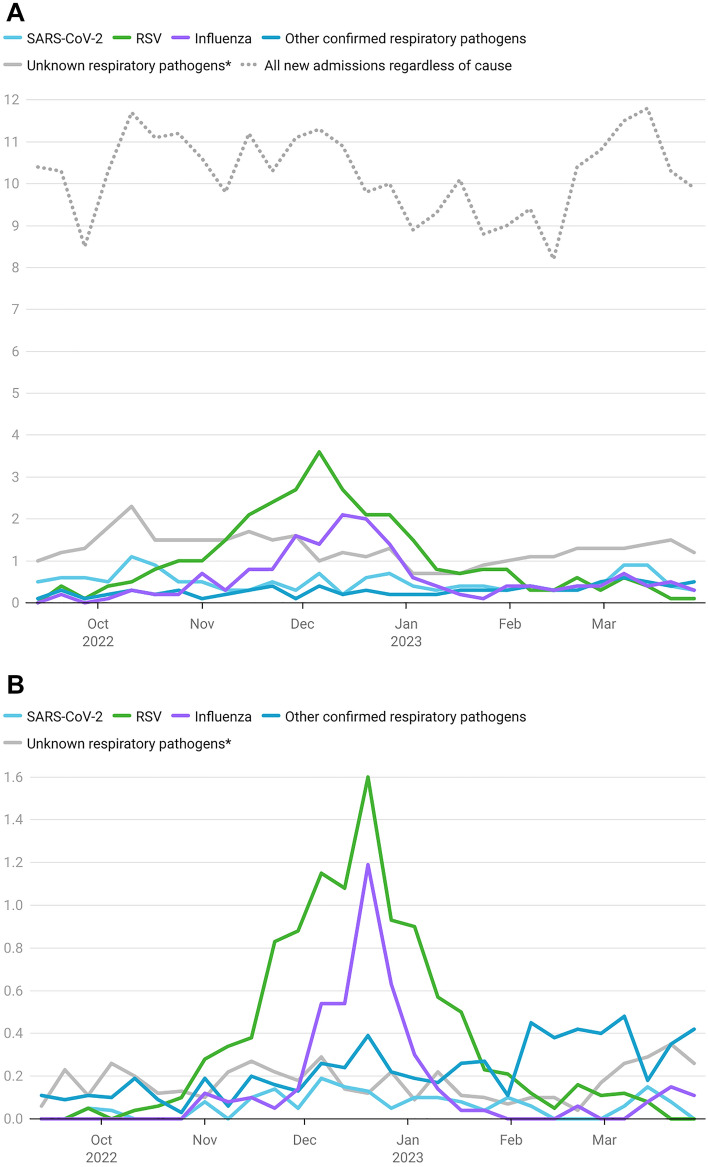
**A** New hospital admissions with respiratory tract infections, average cases per day per reporting hospital. Included in the data analysis were patients who had been newly admitted due to a clinical respiratory infection and whose primary diagnosis also was an acute respiratory infection. **B** Number of patients receiving treatment on ICU due to a respiratory infection, average cases per day per reporting hospital with an in-house ICU. *Pathogen detection pending/not performed

An average of 372 patients per reference day (Tuesdays) were hospitalized, with a peak of 580 absolute cases per day occurring on December 13, 2022. Given the varying rates of survey participation over time, average cases per reporting hospital per reference day were calculated in order to improve comparability. Figure 1A shows the relations and dynamics of each pathogen group leading to hospital admissions and their proportions in comparison to the average number of all hospitalizations regardless of admission diagnosis.

At 50% of total admissions (5.7 out of 11.3 aNA), ARI admissions reached their peak on December 6, 2022. Shortly after this, the proportion of ARI and aNA began decreasing, with just 24.9% (2.2 aNA) being recorded on January 3, 2023. After this point, the proportion of ARI admissions remained < 25%, while the aNA varied from 1.7 to 2.9. The peak in reported RSV and Influenza cases, (3.6 RSV aNA on December 6, 2022, and 2.1 influenza aNA on December 13, 2022), coincided with the peak in reported ARI admissions.

A median of 0.8 patients (range 0.2–2.9 aICU) received ICU treatment in connection with an ARI as a primary diagnosis (Fig. 1B). Two weeks after the aNA apex (December 20, 2022), the average number of ARI cases on ICU peaked at 2.9 aICU, with RSV (1.6 aICU) and influenza cases (1.2 aICU) reaching their highest point. With a median of 0.5 aNA (range 0.2–1.1) and 0.1 aICU (range 0.0–0.2), SARS-CoV-2 played only a minor role in comparison to other respiratory pathogens (Fig. 1).

Commonly reported pathogens included Rhinovirus, Enterovirus, Bocavirus, Adenovirus, human Metapneumovirus, and Parainfluenza virus, as well as bacterial pathogens, such as *S. pneumoniae*, *S. pyogenes*, *H. influenzae*, *S. aureus*, *Pseudomonas aeruginosa* and *Klebsiella pneumonia*. A full itemization of all respiratory pathogens unfortunately cannot be provided due to limitations of the study design. Coinfections occurred in 12.4% among newly admitted patients (n = 112 of all reports) and in 24.3% of ICU patients (*n* = 93 of all reports). Unknown pathogens, (e.g., cases with pathogen detection pending or not performed), were present in 39% (*n* = 1145) of all newly admitted patients, despite the fact that ARI had been listed as their primary diagnosis upon hospital admission.

### Age groups

**Fig. 2:**
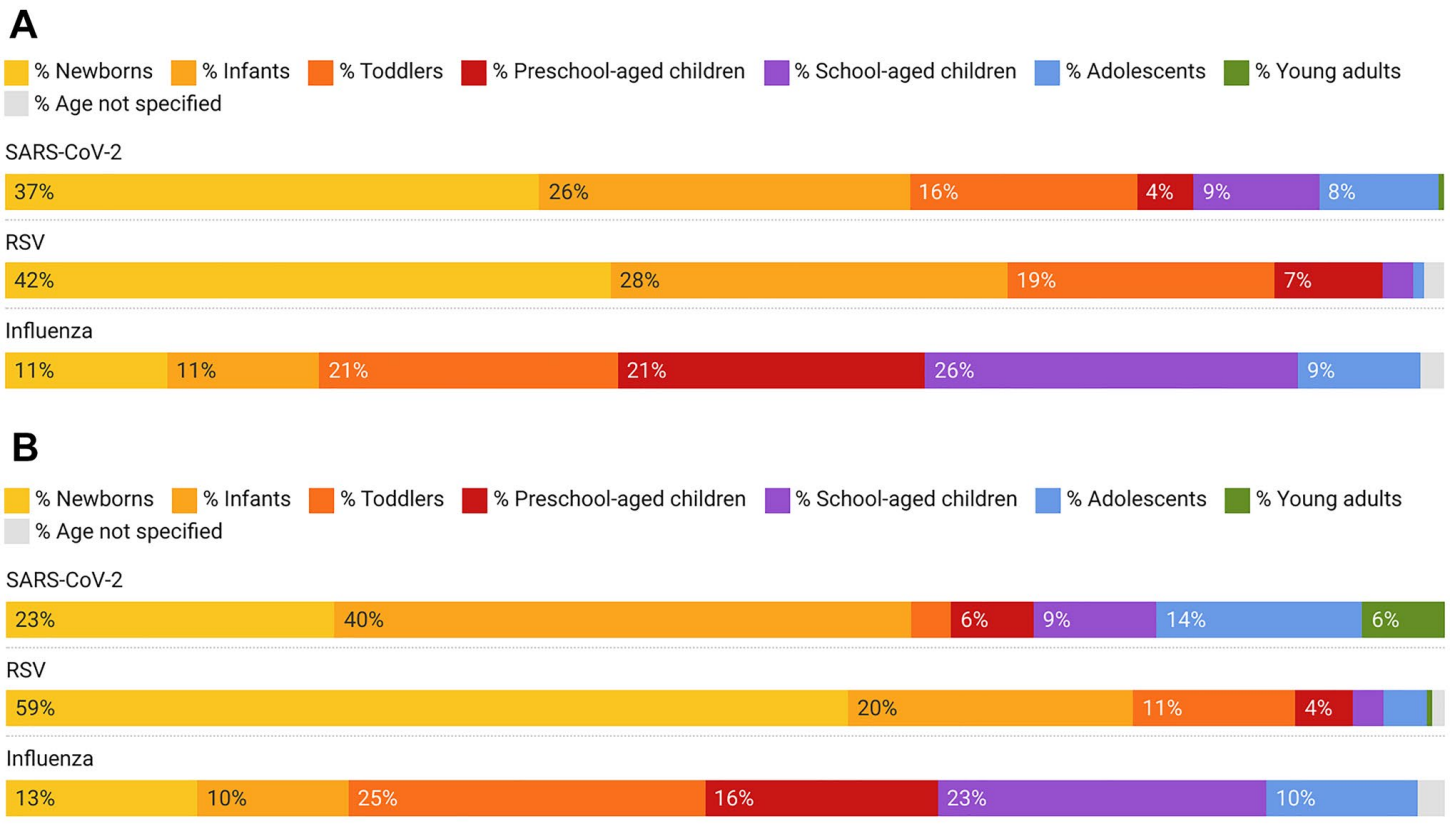
**A** New admissions due to respiratory infections, by age group. **B** Intensive care treatment due to respiratory infections, by age group. Age groups were defined as follows: Newborns = 0–3 months; Infants = 4–11 months; Toddlers = 1–2 years; Preschool-aged children = 3–4 years; School-aged children = 5–11 years; Adolescents = 12–18 years; Young adults ≥ 19 years

Table 1 and Fig. 2 display the distribution of different pathogens according to patient age group. A preponderance of newly admitted patients with RSV and SARS-CoV-2 were newborns or infants. By contrast, the proportion of preschool- and school-aged children was the highest among patients with influenza. For newly admitted patients < 5 years old, the RSV group had the largest proportion of cases (95.7%, *n* = 962/1005); SARS-CoV-2 had the next-largest share, with 82.9% (*n* = 209/252). However, the proportion of < 5-year-olds on ICU followed a slightly different pattern. Here, RSV rates were 93.6% (*n* = 307/328), while those for SARS-CoV-2 were 71.4% (*n* = 25/35) and those for Influenza were 64.7% (= 68/105).

### Comorbidities

In the CLRS, providing information regarding preexisting comorbidities was optional. For this reason, the denominator sizes vary and are not identical with the absolute numbers of patients reported on ICU (Table 1). For ICU patients, we received additional information regarding the presence of comorbidities in 66.3% of cases (*n* = 527/795). In 50.9% of these cases (*n* = 268/527), a significant comorbidity was reported. With respect to specific pathogens, the highest proportion of preexisting comorbidities was reported in the influenza group (62.0%, n = 44), followed by the SARS-CoV-2 group (53.6%, *n* = 15). The RSV group (33.9%, *n* = 63) had the lowest number of reported preexisting conditions (Table 1).

### Respiratory support

In the CLRS, providing information on respiratory support, (defined as invasive or non-invasive ventilation such as with CPAP or high-flow oxygen), also was optional. Therefore, denominator sizes again vary for each group (Table 1). Among ICU patients with ARI, information on respiratory support was submitted in 87.8% of cases reported (*n* = 698/795). Of these, respiratory support was provided to 84.0% of patients (*n* = 586/698). The need for respiratory support was the highest in the RSV group (91.7%, n = 265/289), with the majority of these cases requiring non-invasive ventilation (including CPAP or high-flow oxygen) (87.2%, *n* = 231/265*).* SARS-CoV-2 patients required respiratory support in 63.3% of cases (*n* = 19/30), with 9 (30.0%) needing invasive ventilation. After SARS-CoV-2 patients, influenza patients were the second highest in the proportion receiving invasive ventilation.

## Discussion

In spring 2022, a shift in pandemic-related, non-pharmaceutical public health measures took place in Germany, with requirements for quarantining and mask-wearing becoming loosened. Under these changed circumstances, the CLRS's revised aim became to determine the disease burden of ARI and other select respiratory viruses on pediatric hospitalizations during fall/winter 2022–2023. Although the Robert Koch-Institute, Germany's public health institution, collects extensive data on infection rates and dynamics of various pathogens in the German population [10], their data does not actually provide information on the burden of pathogens leading to pediatric hospitalization. This was the gap our CLRS hoped to fill. By gathering data on and then comparative measuring newly admitted pediatric patients regardless of cause, our CLRS was able to collect information critical to disease burden assessment and related needs for resource allocation. With up to 50% of all admissions having been due to ARI in early December 2022, the combination of the unusually early RSV wave, along with the similarly uncommon, early influenza season, resulted in a significant shortage of hospital beds, with nearly all available pediatric admissions beds getting used for ARI-related admissions. This represented an especially high burden on the hospital system. With our updated CLRS, we hoped to have put in place a tool that would allow us to better understand, and therefore also improve, these circumstances.

In previous pre-pandemic years, RSV season in Germany typically had started in November and had peaked in February/March [11]. In 2021, however, an unusually early and intense RSV season already had taken off by late summer/early fall 2021, with RSV cases reaching their highpoint by the beginning of November 2021 [3, 4]. Interestingly, and to some extent unexpectedly, the 2022/2023 RSV season experienced a sharp rise in cases by early November. Despite a return to a more-or-less typical starting date, the abrupt and significant increase in RSV cases—one even more pronounced than in 2021/2022—seemed surprising. Although the peak number of RSV-related admissions during the 2021/2022 and 2022/2023 RSV seasons was not dramatically different from each other, the number of patients admitted to ICU for RSV infections in 2022/2023 nearly doubled over that of the previous year. By February 2023, however, RSV-related ICU admissions had returned to a level similar to that seen in February 2022 (Supplement S1).

Although RSV predominantly, and almost exclusively, affected under five-year-olds, it nonetheless accounted for the largest proportion of hospital admissions and ICU hospitalizations during the 2022/2023 ARI season. Furthermore, the RSV group displayed the lowest comorbidity rate (33.9%) of any pathogen group in our register—a data point confirming that RSV does not require any specific constitutional weakness in order to succeed in making an infant or young child quite sick. This underlines the importance of developing a safe and effective RSV vaccine for young children, as vaccination could significantly reduce the burden of ARI leading to admissions in pediatric hospitals.

In contrast to ICU patients with RSV, a much higher proportion of ICU patients with influenza (33.3%) and/or SARS-CoV-2 (28.6%) were > 5 years old. For ICU patients, the comorbidity rates reported in the influenza group (62.0%) and SARS-CoV-2 group (53.6%), was higher than that reported in the RSV group (33.9%). This suggests that an influenza and SARS-CoV-2 infection prevention strategy for children > 5 years old is warranted, especially for those where comorbidities may be present.

Despite the easing of public health measures, our CLRS data for 2022/2023 ARI season did not show there to have been a major increase in SARS-CoV-2-related hospitalizations at German pediatric hospitals in relation to this time period—a development already anticipated by our earlier, CLRS-related publication describing the disease burden during spring/summer 2022 when the Omicron variant was predominant [6]. Data provided by the RKI show a similarly low proportion of SARS-CoV-2 on severe ARI during the winter months, especially among under four-year-olds [10]. On this basis, and assuming that no virulence-increasing mutations will develop in the near future, it appears unlikely that SARS-CoV-2 will pose a significant disease burden for children and adolescents in Germany during the upcoming 2023–2024 season. By contrast, the disease burden caused by RSV and influenza could be expected to become as high as in the previous season since there is no reliable way of predicting if the population-based immunity has reached pre-pandemic levels again.

### Limitations

Although there is common agreement in the German pediatric health care community that collecting and analyzing data such as that gathered by our CLRS is important, data reporting can be time-consuming. Without dedicated hospital resources for this purpose, and in the absence of mandatory reporting, regularity and consistency of data collection may be impacted.

Our analysis is additionally limited by the survey design itself. The CLRS's main goal was to measure the burden of ARIs on German pediatric hospitals, not their impact on individuals. For this reason, the CLRS was built to capture anonymized data on ARI-related hospital and ICU admissions over time. The CLRS was not designed to capture individual patient data, e.g., clinical presentation, risk factors, or vaccination status. Although such data of course would have been of significant interest, it could not be analyzed, because it was not captured. Furthermore, we are unable to provide information on ARI admissions or ICU treatment from hospitals that did not participate in the CLRS. Accordingly, we cannot compare data from these hospitals with those that did participate. Therefore, we cannot speculate regarding any potential reporting or selection bias. Although our CLRS did not capture data on types and combinations of coinfections and "complicated respiratory tract infections", on a clinical basis, it was widely reported that these had occurred in high numbers, especially toward the end of 2022 and in early 2023 [12–16]. Unfortunately, these too cannot be shown by means of our CLRS due to its design limitations.

## Conclusion

With 22.8% of German pediatric hospitals contributing data to our CLRS, our study provides a unique opportunity to quantify and concretely evaluate the dimensions of pediatric hospital and disease burden described. In November–December 2022, hospitals participating in our CLRS reported a particularly significant increase in patients with ARI, with these accounting for 50% of all pediatric hospital admissions. RSV and influenza infections represented the greatest proportion of these cases, with an unusual simultaneous incidence peak for both pathogens. Relatively speaking, SARS-CoV-2 played a more minor role, both in terms of absolute numbers and disease severity. These findings underline the importance of conducting ongoing, dynamic, systematic surveillance of ARI data, (as well as that for other infectious diseases), in order to monitor real burdens on the hospital-based health care system. Our CLRS provides a valuable tool for monitoring and assessing pediatric hospital care resources in Germany, and may help in evaluate the future allocation of health care resources here as well. The high voluntary participation rates our CLRS achieved among hospitals, as well as the high quality of data submitted, are critical components for any comparable CLRS. In future, developing systems to automize and systematize data collection in order to preserve staff resources, as well as to provide an even more comprehensive surveillance of ARI epidemiology in children, will provide substantial benefit to the health care system. It also would represent a milestone for future pandemic preparedness as outlined by the WHO.

### Supplementary Information

Below is the link to the electronic supplementary material.

Supplementary file1 (PDF 481 KB)

## Data Availability

Data is shared on reasonably request to the corresponding author.

## Abbreviations

aICU: Average number of patients on ICU who had an ARI as their main diagnosis, per reporting hospital per day
aNA: Average number of newly admitted patients per reporting hospital per day
ARI: Acute respiratory infection
CLRS: Clinician-led reporting system
COVID-19: Coronavirus Disease 2019
CPAP: Continuous positive airway pressure
DGKJ: German Society of Pediatrics
DGPI: German Society for Pediatric Infectious Diseases
ICU: Intensive care unit
REDCap: Research Electronic Data Capture
RKI: Robert Koch-Institute
RSV: Respiratory Syncytial Virus
SARS-CoV-2: Severe Acute Respiratory Syndrome Coronavirus 2
