# Supplementary material for: High burden of acute respiratory tract infections leading to hospitalization at German pediatric hospitals: fall/winter 2022–2023
Source: Infection. 2023 Nov 13;52(2):525–34. doi: 10.1007/s15010-023-02123-7 (PMC10954864; doi:10.1007/s15010-023-02123-7)

Supplement S1: Comparison of average RSV cases per day per reporting hospital, season 2021-2022 vs. season 2022-2023

A: Average number of new RSV admissions, average cases per day per reporting hospital. **B**: Average number of RSV patients receiving ICU treatment, average cases per day per reporting hospital with an in-house ICU.

A.

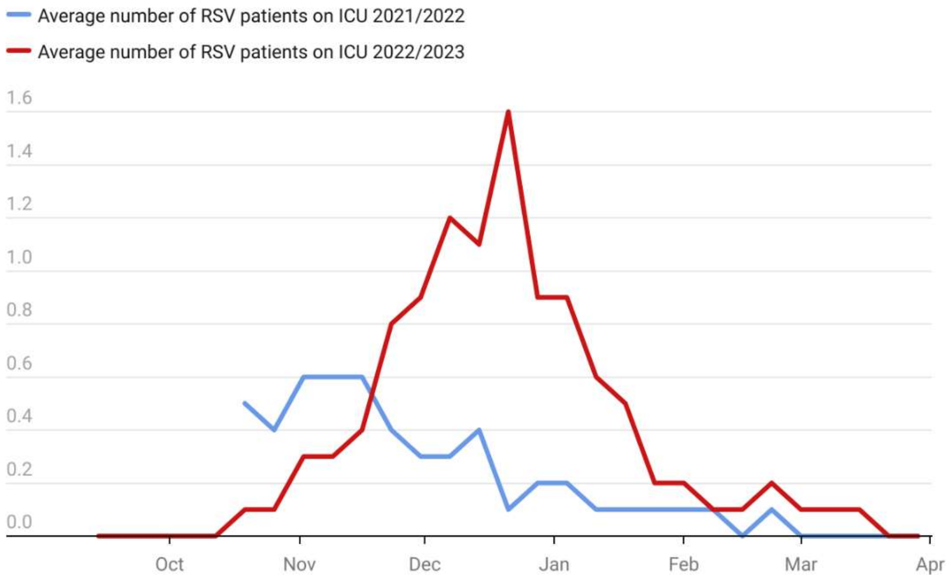

B.

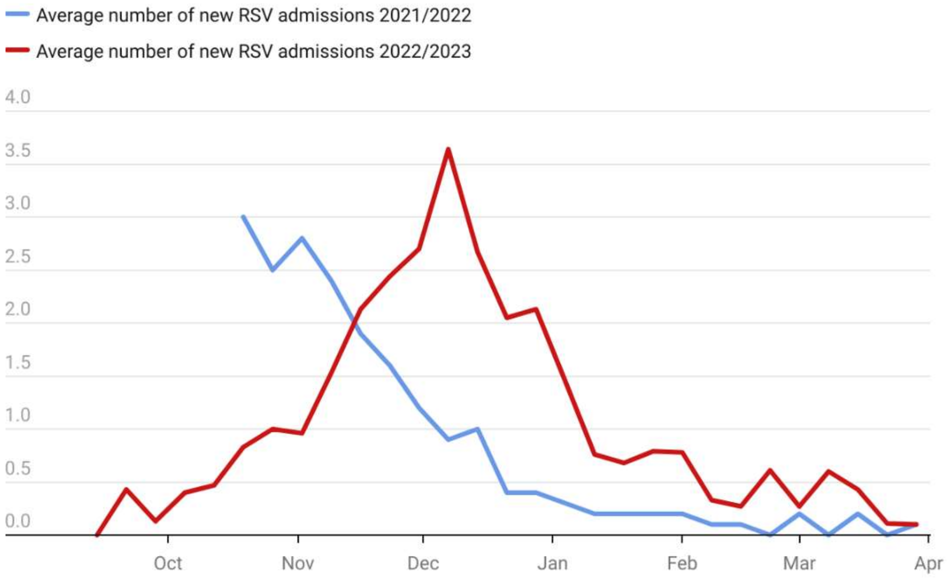

Supplement: Supplementary file 1 — Supplementary file1 (PDF 481 KB) [file 15010_2023_2123_MOESM1_ESM.pdf]
